# Stacked Central Configurations for the Spatial Nine-Body Problem

**DOI:** 10.1155/2013/798468

**Published:** 2013-06-13

**Authors:** Su Xia, Deng Chunhua

**Affiliations:** Faculty of Mathematics and Physics, Huaiyin Institute of Technology, Huai'an 223003, China

## Abstract

We show the existence of the twisted stacked central configurations for the 9-body problem. More precisely, the position vectors *x*_1_, *x*_2_, *x*_3_, *x*_4_, and *x*_5_ are at the vertices of a square pyramid Σ; the position vectors *x*_6_, *x*_7_, *x*_8_, and *x*_9_ are at the vertices of a square Π.

## 1. Introduction and Main Results

The classical *n*-body problem [1, 2] concerns the motion of *n* mass points moving in space according to Newton's law:

(1) $$\begin{array}{c} m_{i}{\ddot{x}}_{i}=-\sum_{j=1,j\neq i}^{n}\frac{m_{i}m_{j}\left(x_{i}-x_{j}\right)}{r_{ij}^{3}},\;i=1,2,\ldots ,n. \end{array}$$

Here, *x*_*i*_ ∈ ℝ^*d*^ is the position of mass *m*_*i*_ > 0, the gravitational constant is taken equal to 1, and *r*_*ij*_ = |*x*_*i*_ − *x*_*j*_| is the Euclidean distance between *x*_*i*_ and *x*_*j*_.

The space of configuration is defined by

(2) $$\begin{array}{c} X=\left\{\left(x_{1},\ldots ,x_{n}\right)\in {\left({\mathbb{R}}^{d}\right)}^{n}:x_{i}\neq x_{j}\;\;\forall i\neq j\right\}, \end{array}$$

while the center of mass is given by

(3) $$\begin{array}{c} c=\frac{m_{1}x_{1}+\cdots +m_{n}x_{n}}{M}, \end{array}$$

where *M* = *m*_1_ + ⋯+*m*_*n*_ is the total mass.

A configuration *x* = (*x*_1_,…, *x*_*n*_) ∈ *X* is called a *central configuration* [2, 3] if there exists a constant *λ*, called the multiplier, such that

(4) $$\begin{array}{c} -\lambda \left(x_{i}-c\right)=\sum_{j=1,j\neq i}^{n}\frac{m_{j}\left(x_{j}-x_{i}\right)}{r_{ij}^{3}},\;i=1,2,\ldots ,n. \end{array}$$

It is easy to see that a central configuration remains a central configuration after a rotation in ℝ^*d*^ and a scalar multiplication. More precisely, let *A* ∈ SO(*d*) and *a* > 0, if *x* = (*x*_1_,…, *x*_*n*_) is a central configuration, so are *Ax* = (*Ax*_1_,…, *Ax*_*n*_) and *ax* = (*ax*_1_,…, *ax*_*n*_).

Two central configurations are said to be equivalent if one can be transformed to the other by a scalar multiplication and a rotation. In this paper, when we say a central configuration, we mean a class of central configurations as defined by the above equivalent relation.

Central configurations of the *n*-body problem are important because they allow the computation of homographic solutions; if the *n* bodies are heading for a simultaneous collision, then the bodies tend to a central configuration (see [3, 4]); there is a relation between central configurations and the bifurcations of the hypersurfaces of constant energy and angular momentum (see [5]).

In this paper, we are interested in spatial central configurations, that is, *d* = 3. In 2005, Hampton [6] provides a new family of planar central configurations for the 5-body problem with an interesting property: the central configuration has a subset of three bodies forming a central configuration of the 3-body problem. The authors [7] find new classes of central configurations of the 5-body problem which are the ones studied by Hampton [6] having three bodies in the vertices of an equilateral triangle, but the other two, instead of being located symmetrically with respect to a perpendicular bisector, are on the perpendicular bisector. The stacked central configurations studied by Hampton [6] were completed by Llibre et al. [8] (see also [9]).

Zhang and Zhou [10] showed the existence of double pyramidal central configurations of *N* + 2-body problem. The authors [11–13] provided new examples of stacked central configurations for the spatial 7-body problem where four bodies are at the vertices of a regular tetrahedron and the other three bodies are located at the vertices of an equilateral triangle.

**Figure 1:**
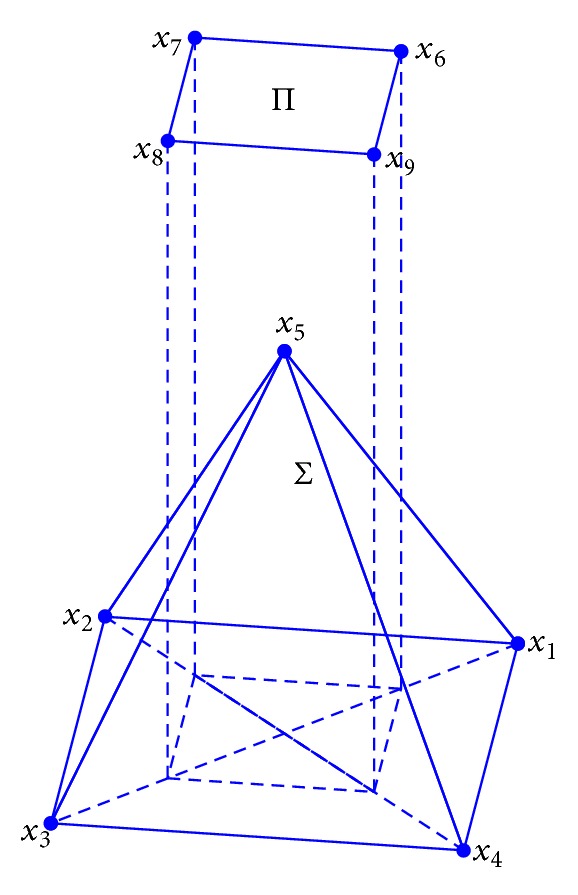
The configuration for the 9-body problem.

In this paper, we find new classes of stacked spatial central configurations for the 9-body problem which have five bodies at the vertices of a square pyramid, and the other four bodies are located at the vertices of a square. More precisely, the spatial central configurations considered here satisfy the following (see Figure 1): the position vectors *x*_1_, *x*_2_, *x*_3_, *x*_4_, and *x*_5_ are at the vertices of a square pyramid Σ; the position vectors *x*_6_, *x*_7_, *x*_8_, and *x*_9_ are at the vertices of a square Π.

Without loss of generality, we can assume that

(5) $$\begin{array}{c} x_{1}=\left(1,0,0\right),\;\;x_{2}=\left(0,1,0\right),\;\;x_{3}=\left(-1,0,0\right),\\ x_{4}=\left(0,-1,0\right),\;\;x_{5}=\left(0,0,h\right),\;\;x_{6}=\left(x,0,y\right),\\ x_{7}=\left(0,x,y\right),\;\;x_{8}=\left(-x,0,y\right),\;\;x_{9}=\left(0,-x,y\right), \end{array}$$

where *x* > 0, *y* ∈ ℝ, and *y* ≠ 0; the positive constant *h* satisfies the equation

(6) $$\begin{array}{c} \frac{2}{r_{15}^{3}}=\frac{1}{r_{12}^{3}}+\frac{1}{r_{13}^{3}}, \end{array}$$

(see [10] and the references therein); that is, *h* = 1.26276522.

The main results of this paper are the following.

**Theorem**

Consider the spatial configurations according to Figure 1, in order that the nine mass points are in a central configuration, the following statements are necessary:

1. the masses *m*_1_, *m*_2_, *m*_3_, and *m*_4_ must be equal;
2. the masses *m*_6_, *m*_7_, *m*_8_, and *m*_9_ must be equal.

**Theorem**

**Figure 2:**
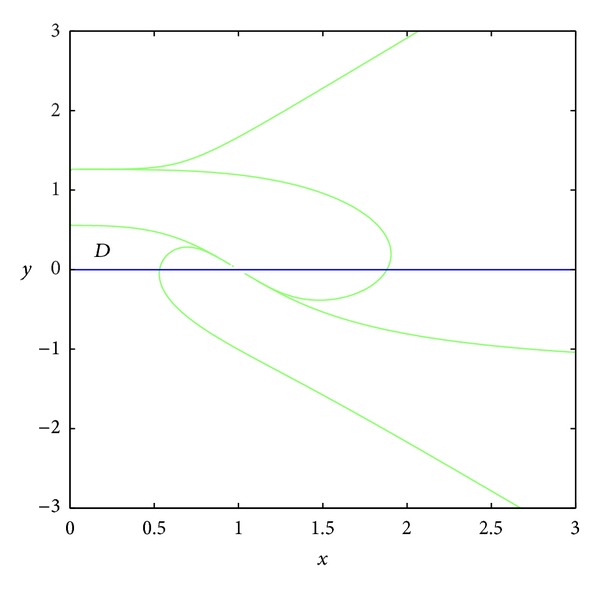
The region *D*.

There exist points (*x*_0_, *y*_0_) ∈ *T* ^−1^(0)∩*D* (see Figure 2) such that the nine bodies take the coordinates

(7) $$\begin{array}{c} x_{1}=\left(1,0,0\right),\;\;x_{2}=\left(0,1,0\right),\\ x_{3}=\left(-1,0,0\right),\;\;x_{4}=\left(0,-1,0\right),\\ x_{5}=\left(0,0,h\right),\;\;x_{6}=\left(x_{0},0,y_{0}\right),\\ x_{7}=\left(0,x_{0},y_{0}\right),\;\;x_{8}=\left(-x_{0},0,y_{0}\right),\\ x_{9}=\left(0,-x_{0},y_{0}\right). \end{array}$$

Then, there are positive solutions of *m*_1_, *m*_5_, *m*_6_ such that these bodies form a spatial central configuration according to Figure 1.

The proofs of the theorems are given in the next sections. 

## 2. Proof of Theorem 1

For the spatial central configurations, instead of working with (4), we consider the Dziobek-Laura-Andoyer equations (see [9, 11–13] and the references therein):

(8) $$\begin{array}{c} f_{ijk}=\sum_{l=1,l\neq i,j,k}^{n}m_{l}\left(d_{il}-d_{jl}\right)\Delta_{ijkl}=0 \end{array}$$

for 1 ≤ *i* < *j* ≤ *n*, *k* = 1,…, *n*, *k* ≠ *i*, *j*. Here, *d*_*ij*_ = 1/*r*_*ij*_^3^ and Δ_*ijkl*_ = (*x*_*i*_ − *x*_*j*_)∧(*x*_*i*_ − *x*_*k*_)·(*x*_*i*_ − *x*_*l*_). Thus, Δ_*ijkl*_ gives six times the signed volume of the tetrahedron formed by the bodies with positions *x*_*i*_, *x*_*j*_, *x*_*k*_, and *x*_*l*_; (8) is a system of *n*(*n* − 1) (*n* − 2)/2 equations.

For the 9-body problem, (8) is a system of 252 equations. According to Figure 1, our class of configurations with nine bodies must satisfy

(9) r12=r23=r34=r14=2,  r13=r24=2,r67=r78=r89=r69=2x,  r68=r79=2x,r16=r27=r38=r49=(x−1)2+y2,r17=r19=r26=r28=r37=r39=r46=r48=x2+1+y2,r18=r29=r36=r47=(x+1)2+y2,r15=r25=r35=r45=1+h2,r56=r57=r58=r59=x2+(y−h)2.

Due to assumption (5) and the definition of Δ_*ijkl*_, we have several symmetries in the signed volumes.

By using the symmetries and the properties of Δ_*ijkl*_, we obtain the following results.

**Lemma**

In order to have a spatial central configuration according to Figure 1, a necessary condition is that the masses *m*_1_, *m*_2_, *m*_3_, and *m*_4_ must be equal.

**Proof**

It is sufficient to consider the equations *f*_687_ = 0 and *f*_796_ = 0:

(10) $$\begin{array}{c} f_{687}=\left(m_{1}-m_{3}\right)\left(d_{16}-d_{18}\right)\Delta_{6871}=0,\\ f_{796}=\left(m_{2}-m_{4}\right)\left(d_{16}-d_{18}\right)\Delta_{7962}=0. \end{array}$$

For our class of central configurations, we have *d*_16_ − *d*_18_ ≠ 0, Δ_6871_ ≠ 0, and Δ_7962_ ≠ 0. So the above equations hold if and only if *m*_1_ = *m*_3_, *m*_2_ = *m*_4_. Consider the expression of *f*_678_ = 0:

(11) f678=(m1−m2)(d16−d17)Δ6781+(m3−m4)(d18−d17)Δ6783=0.

Substituting *m*_1_ = *m*_3_, *m*_2_ = *m*_4_ into the above equation, we have

(12) $$\begin{array}{c} f_{678}=\left(m_{1}-m_{2}\right)\left(d_{16}+d_{18}-2d_{17}\right)\Delta_{6781}=0. \end{array}$$

For our class of central configurations, we have *d*_16_ + *d*_18_ − 2*d*_17_ ≠ 0, since the function *g*(*x*) = *x* ^−3/2^ is convex for all *x* > 0, and Δ_6781_ ≠ 0. So the above equation holds if and only if *m*_1_ = *m*_2_. So statement 1 of Theorem 1 is proved.

**Lemma**

If the configuration, according to Figure 1, is a central configuration, a necessary condition is that the masses *m*_6_, *m*_7_, *m*_8_, and *m*_9_ must be equal.

**Proof**

It is sufficient to consider the equations *f*_132_ = 0 and *f*_241_ = 0:

(13) $$\begin{array}{c} f_{132}=\left(m_{6}-m_{8}\right)\left(d_{16}-d_{18}\right)\Delta_{1326}=0,\\ f_{241}=\left(m_{7}-m_{9}\right)\left(d_{16}-d_{18}\right)\Delta_{2417}=0. \end{array}$$

For our class of central configurations, we have *d*_16_ − *d*_18_ ≠ 0, Δ_1326_ ≠ 0, and Δ_2417_ ≠ 0. So the above equations hold if and only if *m*_6_ = *m*_8_, *m*_7_ = *m*_9_. Consider the expression of *f*_123_ = 0:

(14) f123=(m6−m7)(d16−d17)Δ1236+(m8−m9)(d18−d17)Δ1238=0.

Substituting *m*_6_ = *m*_8_, *m*_7_ = *m*_9_ into the above equation, we have

(15) $$\begin{array}{c} f_{123}=\left(m_{6}-m_{7}\right)\left(d_{16}+d_{18}-2d_{17}\right)\Delta_{1236}=0. \end{array}$$

For our class of central configurations, we have *d*_16_ + *d*_18_ − 2*d*_17_ ≠ 0, and Δ_1236_ ≠ 0. So the above equation holds if and only if *m*_6_ = *m*_7_. Hence, statement 2 of Theorem 1 is proved.

The proof Theorem 1 is completed.

We restrict the set of admissible masses to *m*_1_ = *m*_2_ = *m*_3_ = *m*_4_ = *α* and *m*_6_ = *m*_7_ = *m*_8_ = *m*_9_ = *β*. Substituting *m*_1_ = *m*_2_ = *m*_3_ = *m*_4_ = *α* and *m*_6_ = *m*_7_ = *m*_8_ = *m*_9_ = *β* into (8), they reduce to the following 4 equations:

(16) f152=β((d16+d17−2d56)Δ1526+(d17+d18−2d56)Δ1528)=0,

(17) f162=α(d12+d13−d17−d18)Δ1623+m5(d15−d56)Δ1625+β(d17+d18−d67−d68)Δ1628=0,

(18) f175=α((d12−d16)Δ1752+(d13−d17)Δ1753+(d12−d18)Δ1754)+β((d16−d67)Δ1756+(d18−d67)Δ1758+(d17−d68)Δ1759)=0,

(19) f562=α((d15−d16)Δ5621+(d15−d18)Δ5623+(d15−d17)Δ5624)+β((d56−d67)Δ5627+(d56−d68)Δ5628+(d56−d67)Δ5629)=0.

If we write *f*_152_ = *βT* = *β*((*d*_16_ + *d*_17_ − 2*d*_56_)Δ_1526_ + (*d*_17_ + *d*_18_ − 2*d*_56_)Δ_1528_) = 0, it follows that *T* = 0 in order to have central configurations. So in the following, we restrict our central configurations to the set *T* ^−1^(0).

**Lemma**

According to one's assumptions and the set *T* ^−1^(0), (8) is satisfied if (17) and (18) are satisfied.

**Proof**

Under the assumptions (5), we have

(20) $$\begin{array}{c} T=\left(d_{16}+2d_{17}+d_{18}-4d_{56}\right)\left(y-h\right)+hx\left(d_{16}-d_{18}\right)=0; \end{array}$$

that is,

(21) $$\begin{array}{c} 4\left(y-h\right)d_{56}=\left(y-h\right)\left(d_{16}+2d_{17}+d_{18}\right)+hx\left(d_{16}-d_{18}\right). \end{array}$$

Substituting (21) into (19), we obtain the equation *f*_175_ = 0.

Hence in the set *T* ^−1^(0), *f*_175_ = 0 implies *f*_562_ = 0. This completes the proof.

From Lemma 5, in order to study central configurations according to Figure 1 in the set *T* ^−1^(0), it is sufficient to study the following 2 equations:

(22) $$\begin{array}{c} f_{162}=0,\;\;f_{175}=0. \end{array}$$

Denote by *A* = (*a*_*ij*_) the matrix of the coefficients of the homogeneous linear system in the variables *α*, *m*_4_, *β* defined by (22). Thus,

(23) a11=(d12+d13−d17−d18)Δ1623=−2y(122+18−1(x2+1+y2)3/2−1((x+1)2+y2)3/2),a12=(d15−d56)Δ1625=(−y−hx+h)×(1(1+h2)3/2−1(x2+(y−h)2)3/2),a13=(d17+d18−d67−d68)Δ1628=−2xy(1(x2+1+y2)3/2+1((x+1)2+y2)3/2−18x3−122x3),a21=(d12−d16)Δ1752+(d13−d17)Δ1753+(d12−d18)Δ1754=(y−h)(1((x+1)2+y2)3/2−1((x−1)2+y2)3/2)+hx(14+12−1((x−1)2+y2)3/2−1((x+1)2+y2)3/2−2(x2+1+y2)3/2),a22=0,a23=(d16−d67)Δ1756+(d18−d67)Δ1758+(d17−d68)Δ1759=hx2(1((x+1)2+y2)3/2−1((x+1)2+y2)3/2)−x(y−h)×(1((x+1)2+y2)3/2+1((x+1)2+y2)3/2+2(x2+1+y2)3/2−14x3−12x3).

Let $x=\left(\begin{array}{c} \alpha \\ m_{4}\\ \beta \end{array}\right)$. Then in order to get the spatial central configuration as Figure 1, we need to find a positive solution *α*, *m*_4_, *β* of the following system:

(24) $$\begin{array}{c} Ax=0, \end{array}$$

where *T* = 0. 

## 3. The Existence of Spatial Central Configurations

In order to prove the existence of positive solutions of (24) in the set *T* ^−1^(0), it is sufficient to prove that the entries in each row of *A* change the signs. So if the entries of some row of *A* have the same signs, there are no admissible masses such that the bodies are in a central configuration according to Figure 1.

**Proof of Theorem 2**

Since the rank of matrix *A* is two in the set *T* ^−1^(0), there are nontrivial solutions of (24) in the set *T* ^−1^(0).

Now we prove the existence of spatial central configurations according to Figure 1 for some points in the set *D* (see Figure 2). In order to prove the existence of positive solutions of (24) in the set *T* ^−1^(0), the entries *a*_21_, *a*_23_ of the second line in the matrix *A* should have opposite signs. Thus, we consider the following set *D*, where *D* is surrounded by curves *x* = 0, *y* = 0, *a*_21_ = 0, and *a*_23_ = 0.

**Figure 3:**
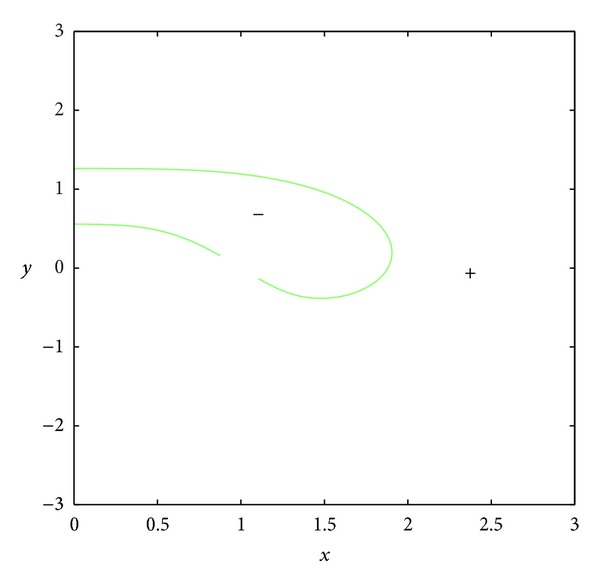
The curve *a*_21_ = 0.

**Figure 4:**
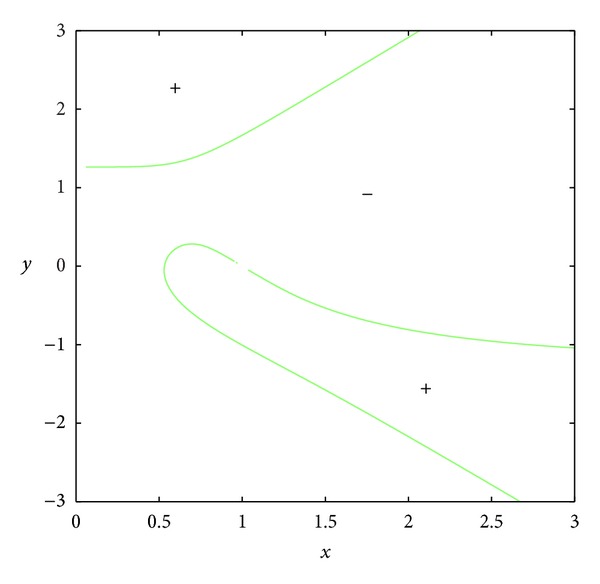
The curve *a*_23_ = 0.

**Figure 5:**
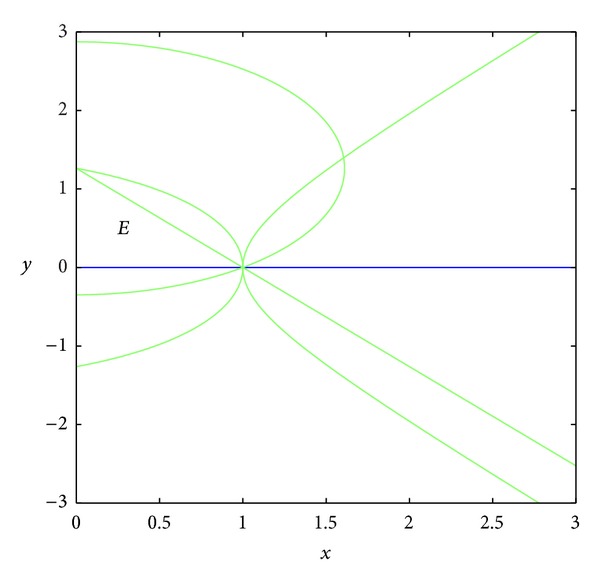
The region *E*.

**Figure 6:**
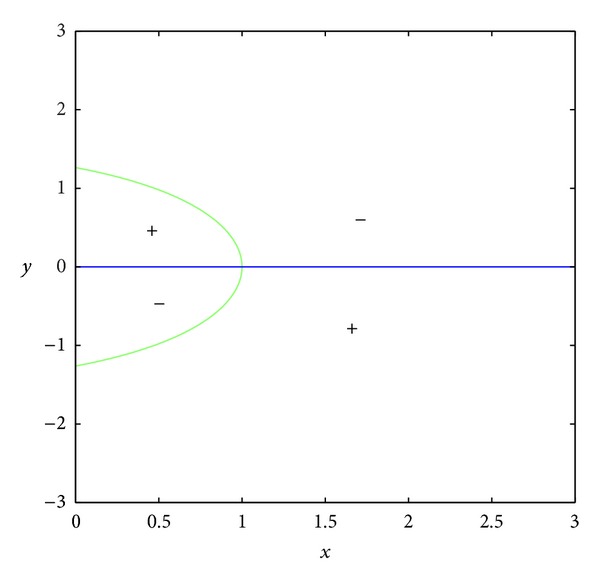
The curve *a*_11_ = 0.

**Figure 7:**
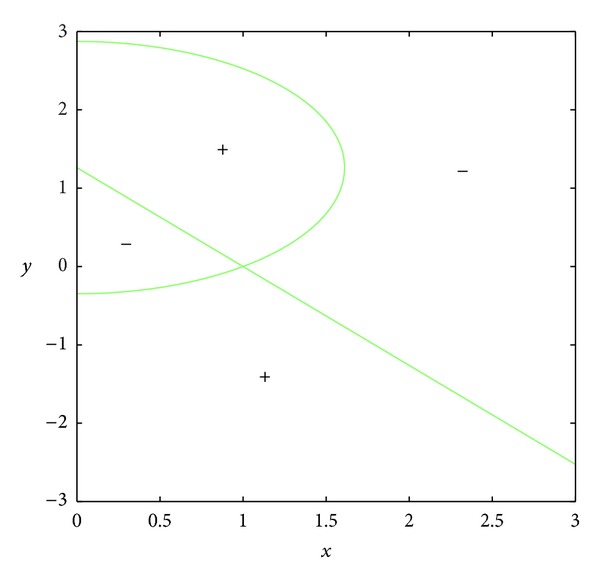
The curve *a*_12_ = 0.

**Figure 8:**
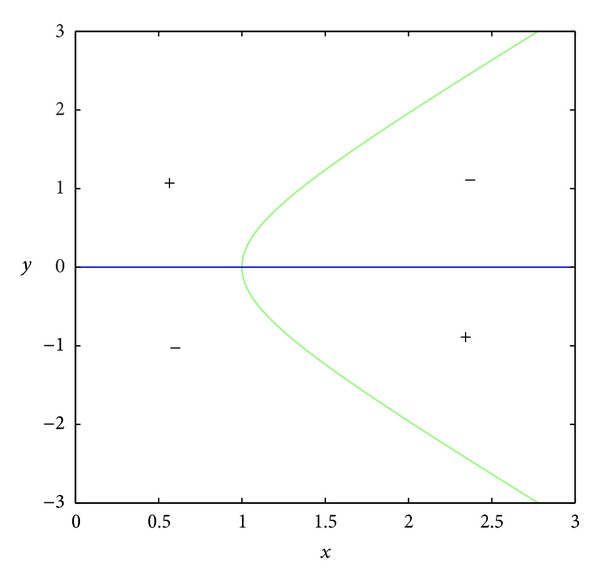
The curve *a*_13_ = 0.

In the set *D*, the entries of matrix *A* have the following signs: *a*_21_ > 0, *a*_23_ < 0 (see Figures 3 and 4); *a*_11_ > 0, *a*_12_ < 0, *a*_13_ > 0 because the set *D* is included in the set *E*, where *E* is surrounded by curves *x* = 0, *y* = 0, and *y* = *h*(1 − *x*) (see Figures 5, 6, 7, and 8). In short, the signs of the entries of the matrix *A* restricted to the set *D* are the following:

(25) $$\begin{array}{c} A=\left(\begin{array}{ccc} + & - & +\\ + & 0 & - \end{array}\right). \end{array}$$

**Figure 9:**
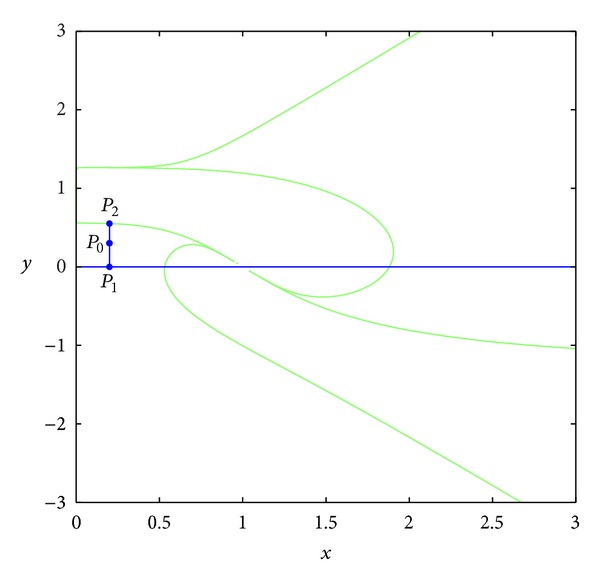
The existence of central configurations for the 9-body problem.

In the rest of the proof, we show that the set *T* ^−1^(0) has intersection with the set *D*. We consider the subset of *D*:

(26) $$\begin{array}{c} L=\left\{\left(x,y\right):x=x_{1},0<y<y_{1}\right\}, \end{array}$$

where *x*_1_ ∈ (0,1). Obviously *L* is a segment with endpoints

(27) $$\begin{array}{c} P_{1}=\left(x_{1},0\right),\;\;P_{2}=\left(x_{1},y_{1}\right), \end{array}$$

(see Figure 9), and the point (*x*_1_, *y*_1_) satisfies the equation *a*_21_ = 0. Evaluating the function *T* at these points, we have

(28) $$\begin{array}{c} T\left(P_{1}\right)<0,\;\;T\left(P_{2}\right)>0. \end{array}$$

Thus, there exists a point *P*_0_ = (*x*_0_, *y*_0_) ∈ *L*, such that *T*(*P*_0_) = 0. So at the point *P*_0_ we have nontrivial positive solutions of (24), since the signs of the entries of the matrix *A* at this point are the following:

(29) $$\begin{array}{c} A\left(P_{0}\right)=\left(\begin{array}{ccc} + & - & +\\ + & 0 & - \end{array}\right). \end{array}$$

Thus, the proof of Theorem 2 is completed.
